# Characterizing a newly identified avian herpesvirus-specific gene SORF3 in DPV and its roles in potential pathogenicity

**DOI:** 10.1128/jvi.01332-25

**Published:** 2025-12-29

**Authors:** Zihang Wang, Huijun Cao, Mingshu Wang, Anchun Cheng, Qiao Yang, Bin Tian, Xumin Ou, Di Sun, Yu He, Zhen Wu, Xinxin Zhao, Ying Wu, Shaqiu Zhang, Juan Huang, Yanlin Yu, Ling Zhang, Renyong Jia, Mafeng Liu, Dekang Zhu, Shun Chen

**Affiliations:** 1 College of Veterinary Medicine, Sichuan Agricultural University, Institute of Veterinary Medicine and Immunology, Research Center of Avian Disease 12529, Chengdu, China; 2 Ministry of Education of the People’s Republic of China, Engineering Research Center of Southwest Animal Disease Prevention and Control Technology, Chengdu, China; 3 Key Laboratory of Animal Disease and Human Health of Sichuan Province, Chengdu, China; 4 International Joint Research Center for Animal Disease Prevention and Control of Sichuan Province, Chengdu, China; University of Virginia, Charlottesville, Virginia, USA

**Keywords:** duck plague, SORF3, specific gene, pathogenic mechanism

## Abstract

Duck plague virus (DPV) is a highly pathogenic avian herpesvirus that affects ducks, geese, and other anseriform poultry. The primary pathological changes observed in infected animals are mucosal, serosal, and systemic hemorrhages accompanied by exceptionally high fatality rates. While most DPV genes are conserved among herpesviruses, a small subset of genes, including the SORF3 gene, is unique to avian herpesviruses. To date, reports on the function and characteristics of the SORF3 gene are limited. In this study, the use of a polyclonal antibody against SORF3 demonstrated that this open reading frame could encode proteins. Through the use of DNA and protein synthesis inhibitors in infected cells, we delineated the gene’s transcriptional and translational timeline, establishing SORF3 as a late gene. To further investigate the role of the protein encoded by the SORF3 gene in the pathogenic mechanism, we constructed a virus lacking the SORF3 gene. The growth kinetics results indicated that the SORF3 protein is not essential for viral replication. *In vivo* experimental findings revealed that while the SORF3-deleted virus still induced clinical symptoms and pathological changes associated with duck plague upon infection of ducks, its lethality was lower than that of the parental virus. In conclusion, this study revealed that the SORF3 gene, which is specific to avian herpesviruses, encodes a late viral protein in DPV and explored its potential role in DPV pathogenesis.

**IMPORTANCE:**

Duck plague virus (DPV) has a high incidence rate and mortality rate of up to 90%, resulting in substantial economic losses in poultry farming. Consequently, investigating the temporal transcription and functional characterization of the proteins encoded by each DPV gene is crucial for understanding its complex life cycle and pathogenesis. This study revealed that the SORF3 gene, identified as an avian herpesvirus-specific gene, encodes a protein. Furthermore, the temporal transcription of this gene throughout the virus’s life cycle confirmed that the protein encoded by SORF3 significantly influences the pathogenicity of DPV.

## INTRODUCTION

Herpesviruses are classified as an ancient family of viruses. Research indicates that approximately 5,000 years ago, during the Bronze Age, herpes simplex virus-1 (HSV-1) disseminated rapidly among populations because of the prevalent custom of kissing (1). Additionally, Marek’s disease virus (MDV) has circulated among poultry in the western regions of Eurasia for at least 1,000 years (2). Herpesviruses comprise three subfamilies: α, β, and γ. Their hosts encompass a diverse array of organisms, including plants, mammals, poultry, fish, and invertebrates. Furthermore, herpesviruses possess substantial genomes that vary in length from 125 to 241 kb, containing between 70 and 170 genes that encode proteins. Notably, 43 of these genes are conserved across the entire herpesvirus family (3). Avian herpesviruses belong to the α-herpesvirus subfamily and represent a distinct category of herpesviruses that exclusively infect poultry (4). The internal regions of avian herpesvirus genomes include unique long sequence regions (ULs), unique short sequence regions (USs), inverted repeat long sequences, and inverted repeat short sequences (IRSs). Different avian herpesvirus genes can be further classified based on the regional locations of their start codons within their genomes; these classifications include LORF, RLORF, SORF, and RSORF, among others (5, 6). The genomes of avian herpesviruses can be divided into two distinct groups. The first group is homologous with other herpesviruses and constitutes the majority of the entire genome, whereas the second group comprises genes unique to avian herpesviruses—referred to as avian herpesvirus-specific genes. Whole-genome sequencing analysis revealed that five avian herpesvirus-specific genes are conserved across all known avian herpesviruses. Among these, four are located in the UL region: LORF2 (vLIP), LORF3, LORF4 (also known as LORF9), and LORF5 (also known as LORF11). The remaining gene, SORF3, is located in the US region (7, 8).

Duck plague (DP), also referred to as duck viral enteritis, is an acute, febrile, and septic infectious disease caused by DP virus (DPV). This disease primarily affects ducks, geese, and other members of the Anatidae family. Infected animals typically display clinical symptoms such as elevated body temperature, anorexia, lethargy, and swelling of the head and neck. Autopsy findings reveal acute septic lesions along with extensive hemorrhaging in various tissues and organs (9). DP is a globally distributed disease characterized by an extremely high fatality rate, resulting in significant economic losses to the poultry industry worldwide. Mature DPV manifests as a spherical granular virus particle characterized by its capsid and cortical structure, measuring approximately 150–300 nm in diameter. As a representative example, the highly virulent CHv strain of DP, identified in China, possesses a typical α-herpesvirus genome organization that follows this sequence from 5′ to 3′: UL-IRS-US-TRS. Genomic sequencing of DPV revealed that its genome comprises a total of 78 open reading frames (ORFs). Among these ORFs are five genes specific to avian herpesviruses: LORF2, LORF3, LORF4, and LORF5, which are located within the UL region, and SORF3, which is located in the US region. Notably, SORF3 is recognized as the sole avian herpesvirus-specific gene found within the US region; it lies adjacent to US2 and US10 and is uniformly designated as SORF3 across all avian herpesvirus species (10).

## RESULTS

### The SORF3 gene of DPV encodes a novel protein

**Fig 1:**
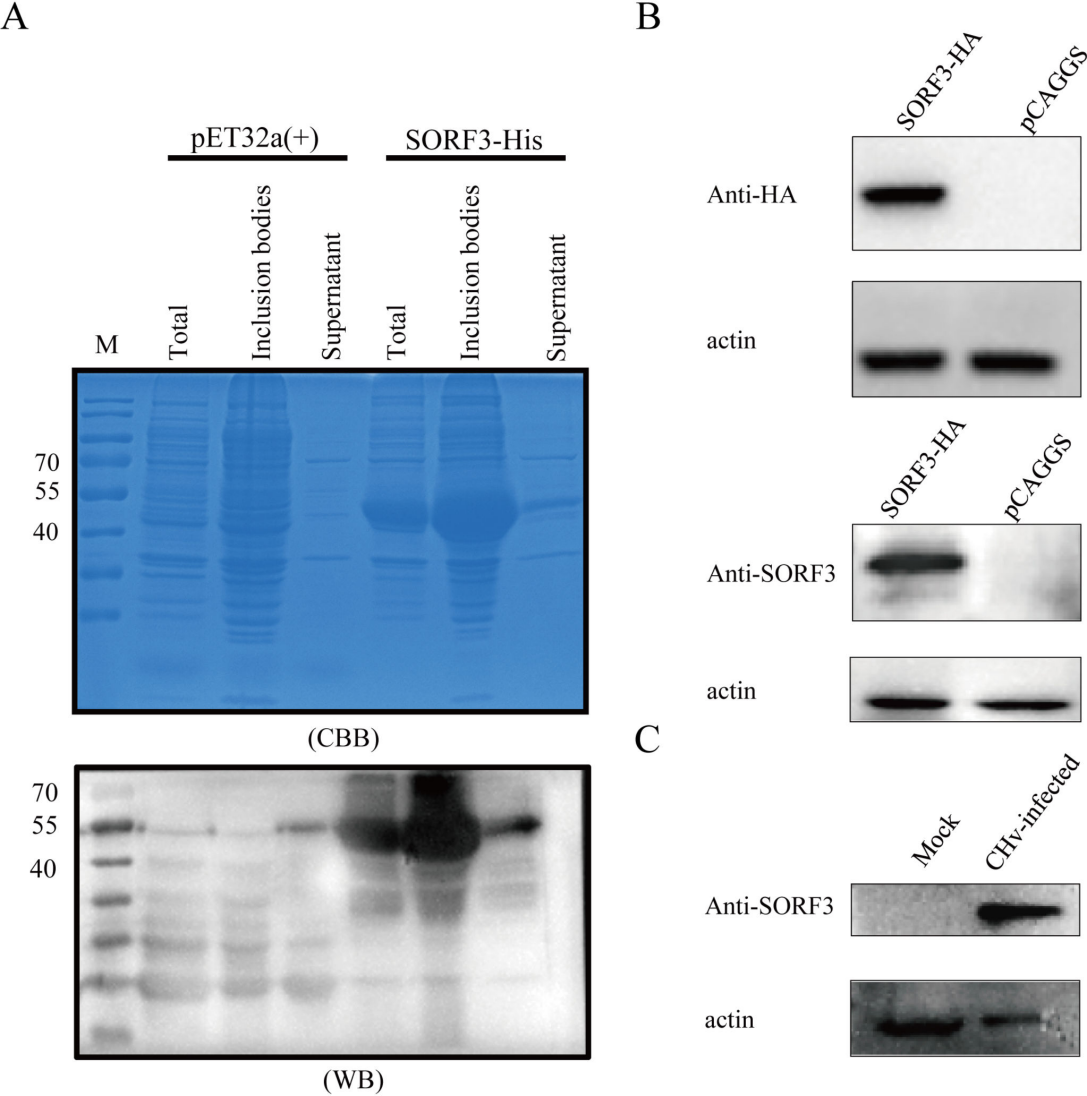
Preparation and expression analysis of DPV SORF3 protein polyclonal antibody. (**A**) The SORF3-His fusion protein was successfully expressed and identified in both soluble and insoluble forms. The SORF3 recombinant protein was expressed via the pET32a vector. Owing to the fusion of thioredoxin (~12 kDa) and hexahistidine (6×His) tags encoded by the vector, the apparent molecular weight of the recombinant protein exceeds the theoretical size of SORF3 alone by approximately 16–20 kDa. (**B**) A mouse anti-SORF3 polyclonal antibody effectively recognized the SORF3 eukaryotic protein through WB. DEF cells were transfected with pCAGGS-SORF3-3HA and harvested 36 h post-transfection. The mouse anti-SORF3 antibody (1:1,000) specifically detected the target protein, while an HA-labeled antibody (1:3,000), along with an empty vector, served as a control. (**C**) WB was used to assess the protein expression levels of SORF3 in cells infected with the virus. DEF cells were infected with a designated virus and collected 24 h after infection, and a mouse anti-SORF3 polyclonal antibody was used for protein detection.

The SORF3-His fusion protein predominantly exists as an expression product in the form of inclusion bodies (Fig. 1A). Following protein enrichment through SDS-PAGE and subsequent gel excision purification, the purified protein was subcutaneously injected into mice via Freund’s complete adjuvant to generate specific anti-SORF3 polyclonal antibodies. The SORF3 protein produced via eukaryotic expression of pCAGGS-SORF3-3HA exhibited specific binding to the polyclonal antibody, with a band size that aligned with the expected molecular weight (Fig. 1B). To further characterize the expression of the avian herpesvirus-specific gene SORF3, we employed the prepared mouse anti-SORF3 antibody for Western blot (WB) analysis. The findings revealed that the protein encoded by the SORF3 gene has an approximate molecular weight of 35 kDa (Fig. 1C). In conclusion, we confirmed for the first time that in DPV, the avian herpesvirus-specific gene SORF3 encodes and expresses a novel protein.

### Construction of recombinant DPV

**Fig 2:**
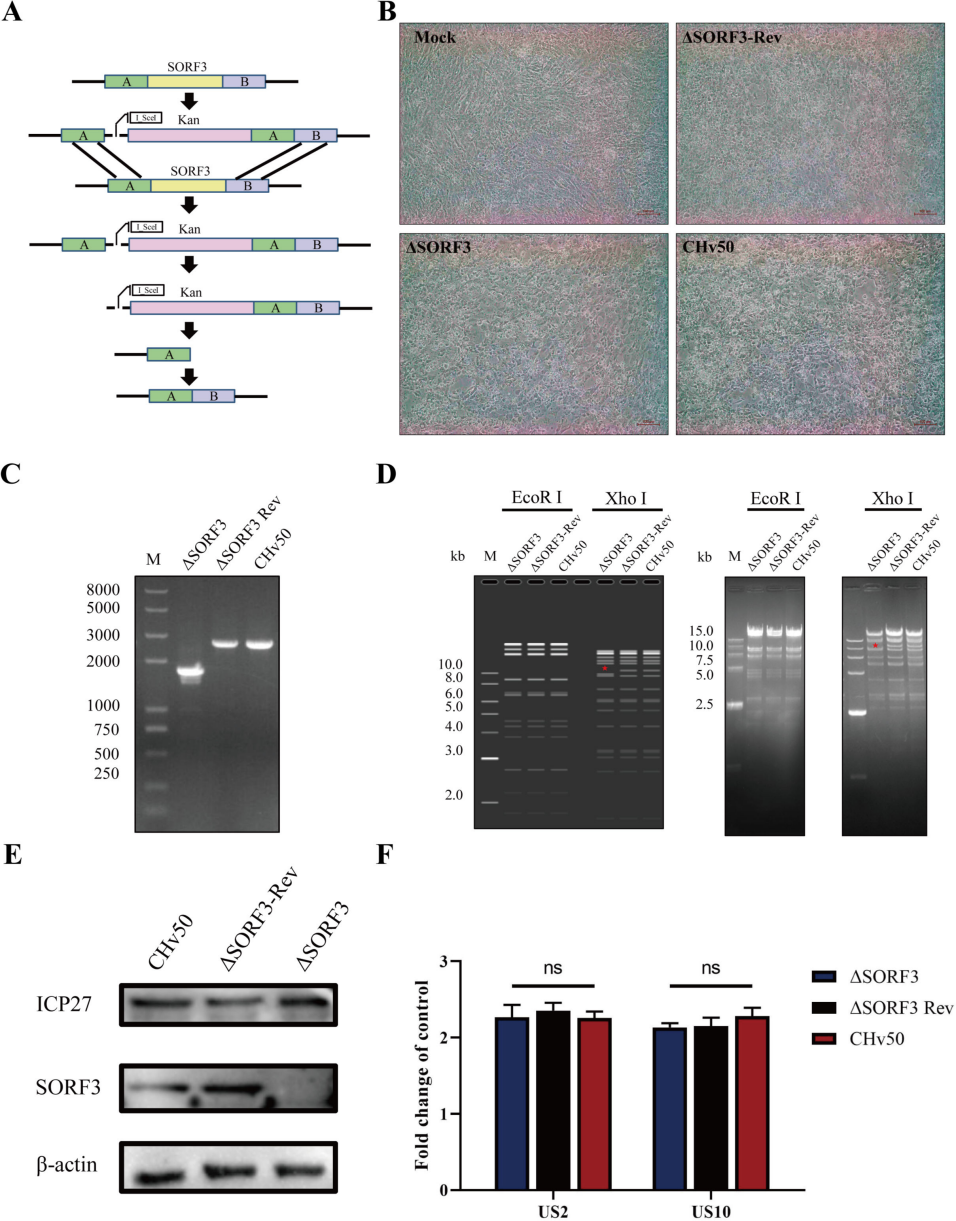
Construction of the SORF3 knockout virus and rescue virus. (**A**) Principle of SORF3 gene knockout. In the initial step, the kanamycin resistance gene (kan) was employed for homologous recombination to replace the SORF3 gene. In the subsequent step, L-arabinose treatment activated I-SceI to cleave its recognition site, thereby facilitating the removal of the kan gene via a second round of homologous recombination, to ensure complete SORF3 deletion from the DP virus genome. (**B**) BAC-free SORF3 deletion mutants (ΔSORF3), reversion mutants (ΔSORF3-Rev), and parental strains (CHv50) were harvested after rescue and purification. (**C**) PCR identification confirms the presence of the recombinant virus. The ORF of the SORF3 gene comprises 888 nucleotide base pairs (bp). The band was amplified via SORF3 gene deletion identification primers (expected size: 1,528 bp), successfully validating the deletion of the SORF3 gene. Lanes 2–3: amplification bands from the wild-type and recovery-type controls (expected size: 2,416 bp), demonstrating the complete SORF3 gene sequence. (**D**) RFLP analysis validating the recombinant virus construction. Following treatment of infectious clone plasmids derived from knockout viruses with QuickCut-Xho I, a band corresponding to 10,000 bp was absent (as indicated in the figure), whereas no differences were observed when QuickCut-*EcoR* I was used. All the experiments depicted in this figure utilized 1% agarose gel electrophoresis imaging techniques. (**E**) WB analysis verifying the construction of the recombinant virus. After the cells were infected with recombinant viruses for 24 h, cell lysis was performed, followed by the collection of samples. The expression levels of the SORF3 protein were then assessed using anti-SORF3 polyclonal antibodies, with β-actin serving as an internal control. (**F**) Assessment of how infection by SORF3 knockout viruses influences the expression levels of upstream and downstream gene (ns indicates *P* > 0.05).

Using a bacterial artificial chromosome recombinant constructed in our laboratory, we successfully employed a two-step red recombination system (Fig. 2A) to construct and rescue the SORF3 deletion mutant DPV-ΔSORF3, as well as the rescue virus DPV-ΔSORF3-Rev. Notably, significant cytopathic effects were observed in cells infected with these viruses compared with those in normal cells (Fig. 2B). Polymerase chain reaction (PCR) analysis via specific primers confirmed that the complete SORF3 gene fragment was present in both DPV-ΔSORF3-Rev and its parental virus DPV-CHv50, whereas it was absent in DPV-ΔSORF3 (Fig. 2C). Furthermore, the RFLP (restricted fragment length polymorphism) results demonstrated that following enzyme digestion with *Xho* I, a nucleic acid band was absent at 10,000 bp for DPV-ΔSORF3 compared with the results for both DPV-ΔSORF3-Rev and DPV-CHv50 (Fig. 2D), which aligned with our predictions. To further determine whether the SORF3 gene had been completely deleted, we verified the expression of the SORF3 protein through WB. ICP27 served as an internal reference for viral proteins, whereas β-actin served as an internal control for cellular proteins. The expression of the SORF3 protein, encoded by the SORF3 gene, was entirely abolished following its deletion (Fig. 2E). These findings collectively validated the successful construction of the deletion virus. Notably, deletion of the SORF3 gene did not affect the expression levels of adjacent upstream and downstream genes (Fig. 2F).

### SORF3 is classified as a late (L) gene

**Fig 3:**
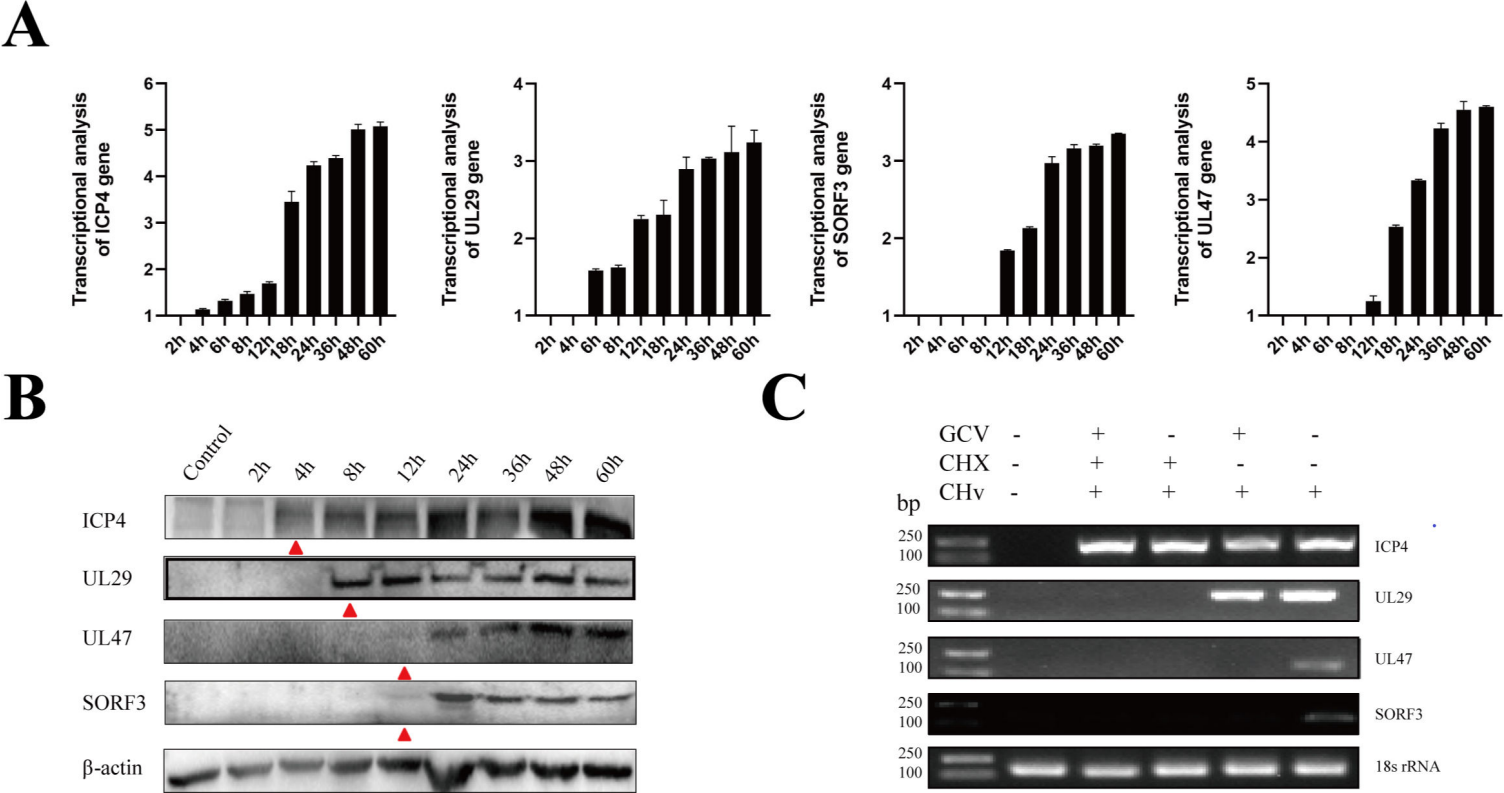
Analysis of SORF3 gene types. (**A**) Total RNA was isolated from both control cells and DPV-infected cells at various time points and then subjected to RT-qPCR analysis. The results were normalized to β-actin, and the fold change relative to the blank control cells was plotted. ICP4, UL29, and UL47 served as controls for IE, E, and L gene expression, respectively. (**B**) Expression analysis of SORF3 gene products in DPV-infected DEFs at different time intervals is presented. The samples and gels from the same experiment were processed simultaneously to ensure consistency. (**C**) Following the infection of cells treated with GCV and CHX with DPV for 18 hpi, the isolated RNA was reverse-transcribed into cDNA, which was then used as a template for nucleic acid gel electrophoresis analysis. The characteristics of the SORF3 gene were identified through the expression patterns of each gene type.

To determine the transcriptional phase of the SORF3 gene, samples were collected at 2, 4, 6, 8, 12, 18, 24, 36, and 48 h post-infection (hpi) with DPV-CHv50 in duck embryonic fibroblasts (DEFs). The transcriptional level of SORF3 was assessed via quantitative reverse transcription PCR. The classification of the SORF3 gene type utilized β-actin as an internal reference control, ICP4 as a control for the immediate-early (IE) gene, UL29 as a control for the early (E) gene, and UL47 as a control for the L gene. The results indicated that the expression of the SORF3 gene aligned with that of the L gene UL47, which was first detected at 12 hpi and presented increasing expression levels over time. In contrast, the expression of both the IE gene ICP4 and the E gene UL29 was detected at 4 h and 8 hpi, respectively, and their expression levels gradually increased over time (Fig. 3A). To further elucidate the classification of the genetic type of SORF3, we examined its expression phase. The results demonstrated that the expression phases of the SORF3 protein closely aligned with those of the UL47 protein; both commenced at approximately 12 h after infection and displayed similar expression profiles (Fig. 3B). Finally, we performed drug inhibition experiments by treating cells with cycloheximide (CHX) or ganciclovir (GCV) individually, while establishing a control group for comparison. There was an absence of detectable bands in both the CHX treatment group and the GCV treatment group, as well as in the combined CHX + GCV treatment group (Fig. 3C). In alignment with the findings observed during the transcriptional and expression phases, these findings suggest that SORF3 functions as a L gene of DPV.

### Distribution of pSORF3 in DEFs

**Fig 4:**
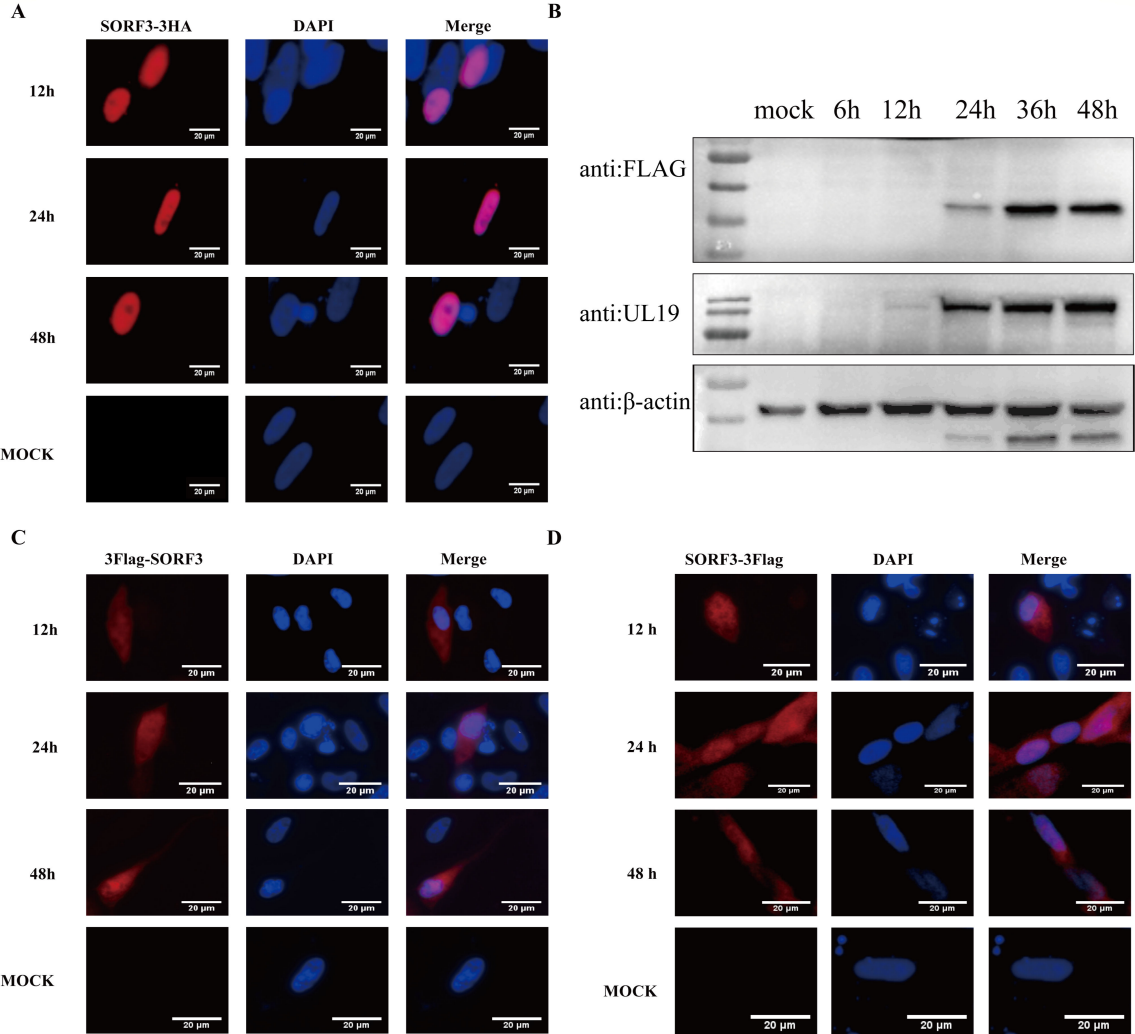
Intracellular localization and distribution of the SORF3-encoded protein. (**A**) Overexpression of pSORF3 in DEFs (depicted in red), including DEF cells transfected with the pCAGGS empty vector, was analyzed via HA-labeled monoclonal antibodies and goat anti-mouse IgG. (**B**) The expression of SORF3-3FLAG in DEFs infected with DPV-BAC-SORF3_C3*Flag_-tagged virus was detected at various time points: 6, 12, 24, 36, and 48 h. (**C**) Infection of DEFs with DPV-BAC-SORF3_N3*Flag_ at a multiplicity of infection (MOI) of 0.1 is shown (highlighted in red), with uninfected cells serving as the control group; detection utilized Flag-labeled monoclonal antibodies and goat anti-mouse IgG. (**D**) Infection of DEFs with DPV-BAC-SORF3_C3*Flag_ at an MOI of 0.1 is shown (highlighted in red), with uninfected cells serving as the control group; detection utilized Flag-labeled monoclonal antibodies and goat anti-mouse IgG.

To investigate the subcellular distribution of pSORF3 in DEFs, we constructed an eukaryotic expression plasmid incorporating an HA tag. Following transfection into DEFs, coverslips were collected at 12, 24, and 48 h post-transfection. The use of an anti-HA monoclonal antibody as the primary antibody for indirect immunofluorescence analysis revealed that the HA-tagged SORF3 protein was specifically localized within the nucleus (Fig. 4A). To further examine the distribution of the SORF3 protein during viral infection, we engineered a tagged virus, DPV-BAC-SORF3_C3*Flag_, in which SORF3 was fused with a FLAG tag. The expression of the SORF3-FLAG fusion protein was successfully detected (Fig. 4B). Similarly, the use of an anti-Flag monoclonal antibody as the primary antibody in immunofluorescence assays demonstrated that the SORF3 protein is uniformly distributed in both the nucleus and cytoplasm, regardless of whether the Flag tag is positioned at the N-terminus or C-terminus of the target protein. (Fig. 4C and D). In summary, when expressed independently, the SORF3 protein was entirely localized to the nucleus. However, under viral infection conditions, there was a shift in its distribution toward the cytoplasm, although it remained primarily concentrated within the nucleus.

### *In vitro* growth kinetics of SORF3 mutants

**Fig 5:**
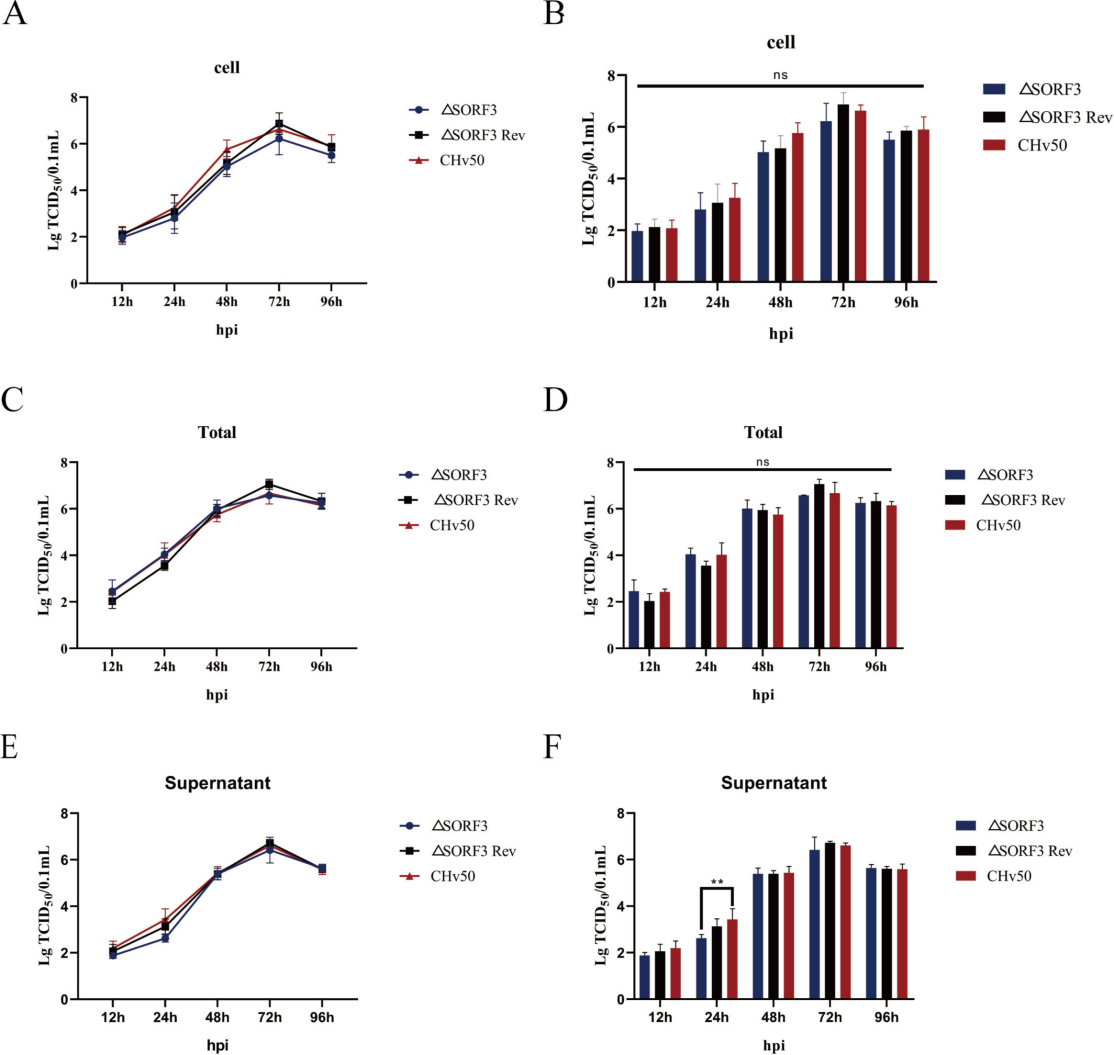
Multistep growth kinetics analysis of the recombinant virus. (**A**) Curve depicting the viral replication levels in the cell samples. (**C**) Curve illustrating the degree of viral replication in the culture medium. (**E**) Curve representing the viral replication levels across all the samples. (**B, D, F**) Statistical analysis of differences in virus titers at each time point for cells (**B**), supernatants (**D**), and total samples (**F**). Asterisks denote significant differences compared with the wild-type (WT) virus. The data are presented as average virus titers from three independent experiments, along with standard deviations for each experiment (ns, *P* > 0.05).

To investigate the role of pSORF3 in DPV replication, we analyzed its *in vitro* growth kinetics. The results indicated that after DEFs were infected with DPV-ΔSORF3, DPV-ΔSORF3-Rev, or the parental virus DPV-CHv50 (WT) at a multiplicity of infection (MOI) of 0.01, all the viruses exhibited comparable proliferation patterns (Fig. 5A through F). As the duration of infection increased, viral particle replication tended to increase, reaching a peak at 72 hpi. Following this peak, viral replication began to decelerate. Data analysis via GraphPad Prism version 8 revealed a significant increase in virus titers within cells, supernatants, and total fluids at the 72 h mark, indicating maximal viral replication during this period, with a substantial number of maturely assembled viral particles present within cells and some being released into the environment. Extracellularly, from 72 to 96 hpi, there was a decrease in virus titers across cells, supernatants, and total fluids. This decrease may be attributed to extensive cytopathic effects occurring during this timeframe, which lead to cell loss and consequently reduced viral presence within remaining cells. Notably, the growth curves for both DPV-ΔSORF3 and DPV-ΔSORF3-Rev were consistent with those observed for the parental virus. Based on these findings, we conclude that the SORF3 protein is not essential for viral replication.

### pSORF3 does not influence the *in vitro* replication of DPV

**Fig 6:**
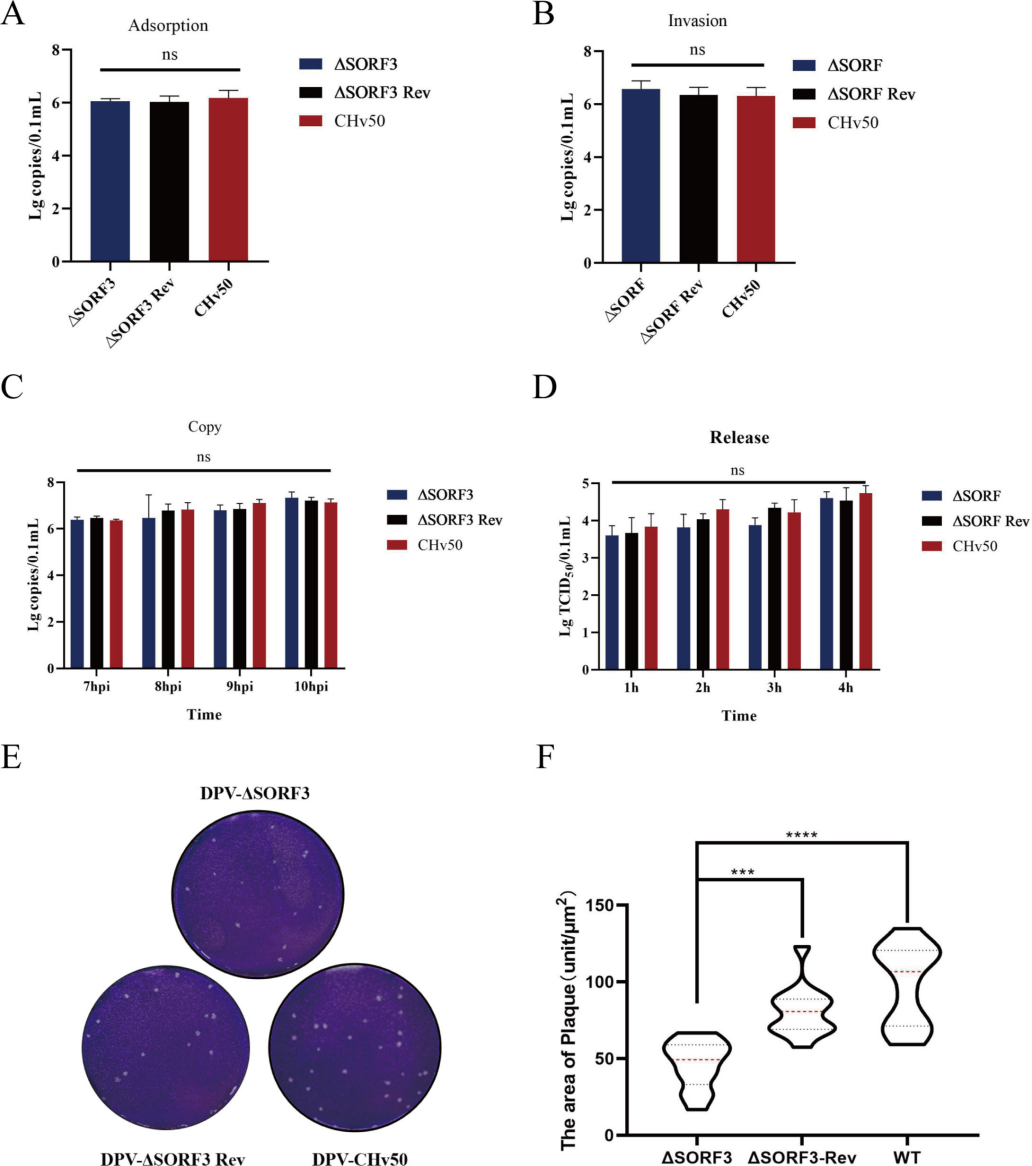
Role of pSORF3 in various stages of viral processes, including adsorption, entry, replication, release, and intercellular transmission. (**A**) DEFs chilled at 4°C (to block endocytosis) were inoculated with viruses. After 2 h of adsorption, the unbound virions were removed by washing with PBS. The cell-associated viral genomes were quantified via qPCR to calculate the adsorption efficiency. (**B**) After adsorption, the cells were shifted to 37°C for 1 h to activate endocytosis. The intracellular viral loads were quantified to evaluate the efficiency of virus entry. (**C**) DEFs infected with DPV (MOI = 1) were replenished with fresh maintenance medium at 6 hpi to remove residual virions. The cells were harvested at 13–16 hpi (7–10 h post-replenishment) for quantification of intracellular viral DNA via quantitative PCR (qPCR). (**D**) Supernatants collected at 1–4 h post-replenishment (19–22 hpi) after the medium was changed at 18 hpi. Infectious virion release was quantified via a TCID_50_ assay (log_10_ TCID_50_/mL). (**E**) Intercellular spread was assessed via the crystal violet staining method. (**F**) Intercellular spread was further evaluated by selecting 10 plaques randomly from each group following crystal violet staining. The plaque areas were scanned and measured via ImageJ software (ns indicates *P* > 0.05; *, *P* < 0.05; **, *P* < 0.01; ***, *P* < 0.001; ****, *P* < 0.0001).

The life cycle of DPV, similar to that of other herpesviruses, encompasses several stages: adsorption, entry, replication, release, and intercellular transmission. To investigate the impact of the SORF3 protein on this viral life cycle, cells were infected with DPV-ΔSORF3, DPV-ΔSORF3-Rev, or the parental virus. We subsequently analyzed the virus copy numbers, titers, and plaque formation results. The experimental data revealed no significant differences in virus copy numbers or titers among the groups infected with DPV-ΔSORF3, DPV-ΔSORF3-Rev, or DPV-CHv50 (WT) during the adsorption, entry, replication, and release assays. These findings suggest that the SORF3 protein may not play a role in early viral infection processes and does not influence the overall life cycle of DPV (Fig. 6A through D). Furthermore, the plaque assay results revealed that plaques formed by DEFs infected with DPV-ΔSORF3 were smaller in both quantity and area than those formed by DPV-CHv50 (WT)- and DPV-ΔSORF3-Rev-infected DEFs (Fig. 6E and F). These findings indicate that expression of the SORF3 gene may facilitate intercellular transmission.

### Deletion of the SORF3 gene significantly diminishes the virulence of DPV

**Fig 7:**
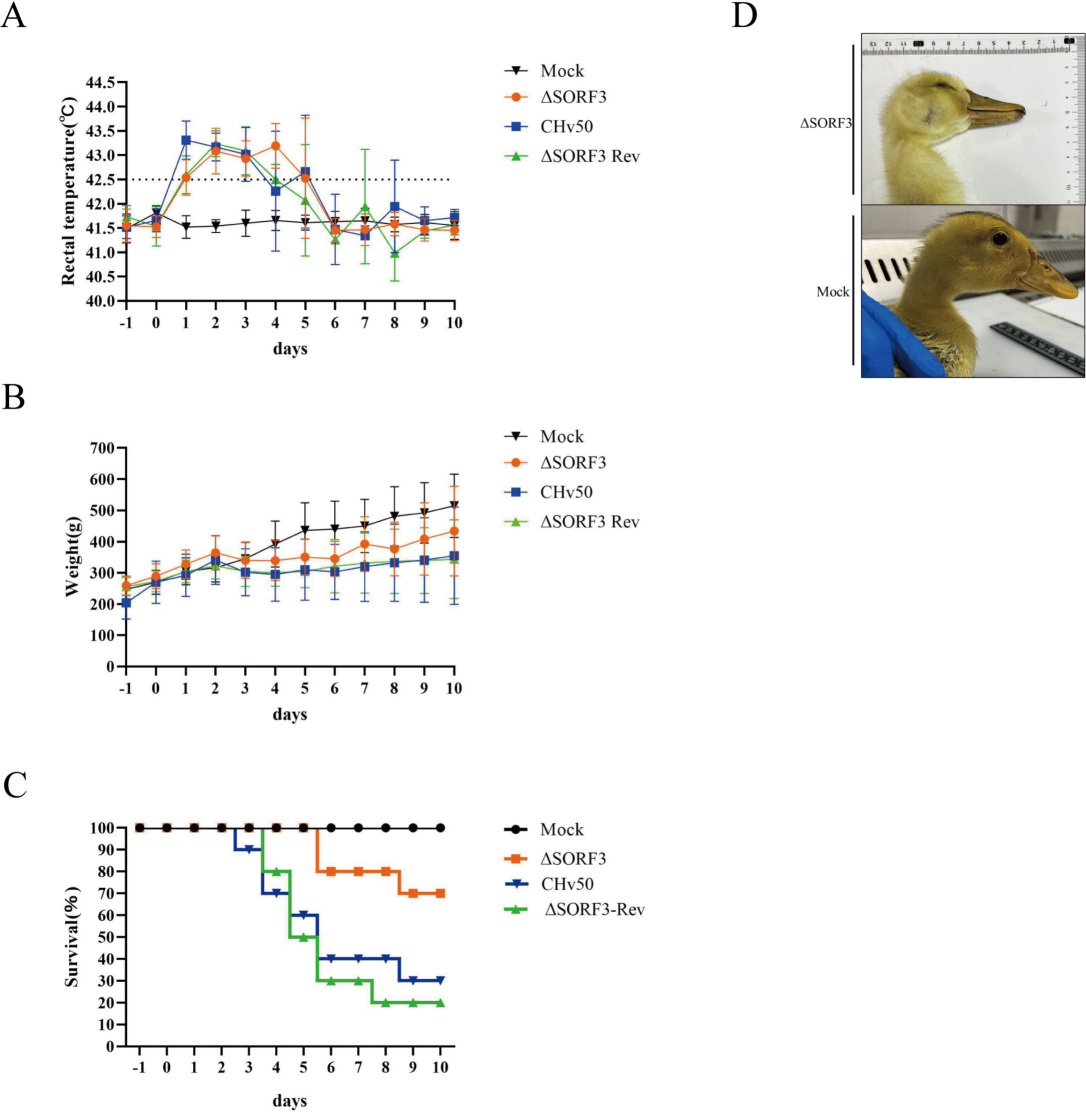
Pathogenesis of the deletion, restoration, and parental viruses. (**A**) Rectal temperature of ducks following inoculation with DPV. Temperature monitoring was conducted on ducklings inoculated with 10^6^ TCID_50_ over 10 days, and the temperature change trends for each group were plotted. (**B**) Weight gain in ducks post-inoculation with DPV. Weight monitoring was performed on ducklings inoculated with 10^6^ TCID_50_ for 10 days, and the weight trend curves are shown. (**C**) Survival curve of ducks infected with DPV. The number of fatalities in each group was recorded daily for 10 days, resulting in survival rate charts for each group being generated. (**D**) Clinical symptoms observed in ducks after infection with DPV-ΔSORF3.

To investigate the involvement of the SORF3 gene in the pathogenesis of DPV, we infected 14-day-old ducklings with DPV-ΔSORF3, DPV-ΔSORF3-Rev, and DPV-CHv50 viruses at a dilution of 10^6^ TCID_50_ and monitored their condition over 10 days. Our observations revealed that ducks in both the group infected with the parental virus and the group infected with the rescue virus presented fever symptoms starting 24 hpi, with maximum temperatures exceeding 43.5°C. In contrast, ducks in the group infected with the SORF3-knockout virus did not display fever until 48 h after infection (Fig. 7A). Body weight monitoring revealed that, compared with those in the mock-infected group, once fever onset occurred in ducks from the parental virus group, the reconstituted virus group, and the deletion virus group, there was a noticeable slowdown or even a decrease in weight gain (Fig. 7B). However, their appetites gradually improved thereafter; by days 7 and 8 post-infection, slight increases in weight were observed. Survival rate assessments for ducks infected with different viruses revealed that by days 7 and 8 following infection with DPV-CHv50, DPV-ΔSORF3-Rev, or DPV-ΔSORF3, and day 10 after DMEM injection (mock infection), survival rates of 30%, 20%, 70%, and 100%, respectively, were detected (Fig. 7C). Furthermore, ducks infected with DPV-ΔSORF3 continued to exhibit symptoms consistent with “big-head disease” (Fig. 7D).

### Deletion of the SORF3 gene mitigates tissue damage caused by DPV

**Fig 8:**
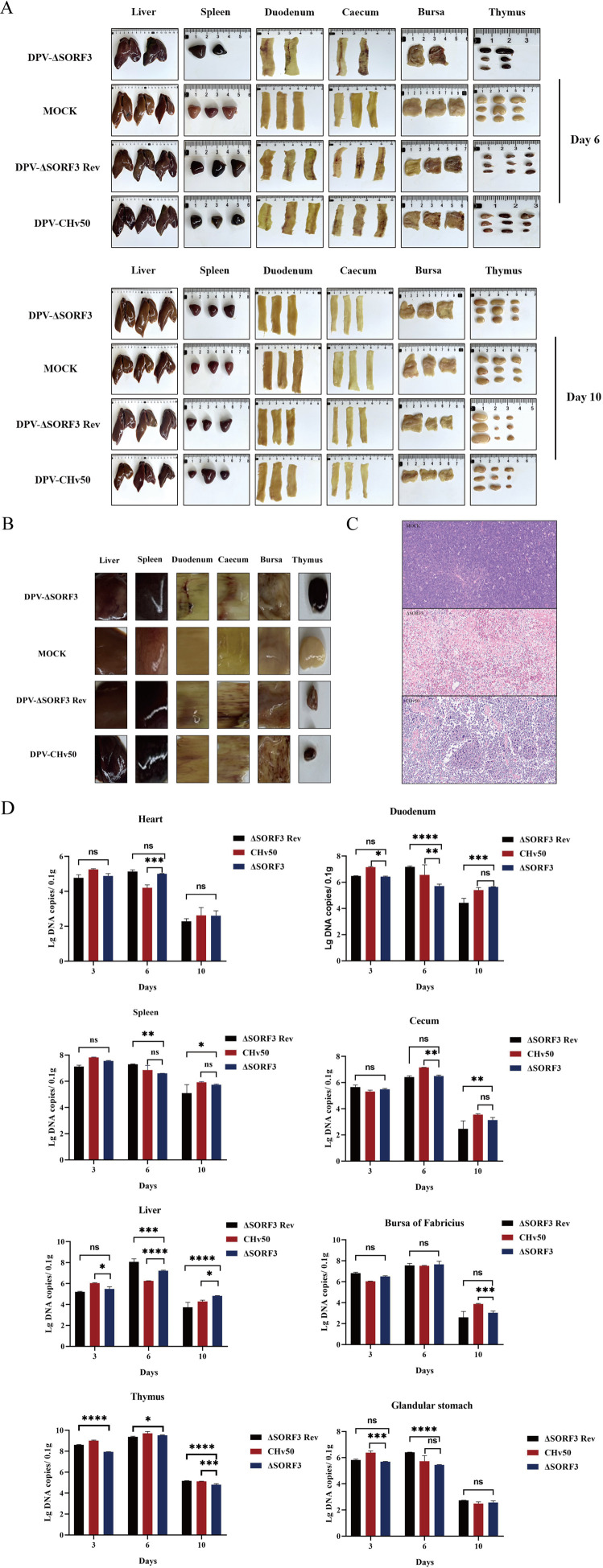
Illustrates the organopathology and microscopic pathology observed in ducks infected with the specified virus. (**A–D**) Documented instances of organopathological damage in infected ducklings from the knockout strain, rescue strain, and parental strain at 6 to 10 days post-infection. (**B**) Magnified view of the local lesion of the organs in the panel. (**C**) Microscopic pathological examination of thymic tissue in ducklings infected with both the knockout strain and parental strain, using MOCK as a control. (**D**) Replication characteristics of SORF3 mutants *in vivo* are presented. A quantitative assessment was conducted to determine the copy number of the DPV genome for the detected virus across various organs, including heart, liver, spleen, thymus, duodenum, cecum, proventriculus, and bursa of Fabricius (ns, *P* > 0.05; *, *P* < 0.05; **, *P* < 0.01; ***, *P* < 0.001; ****, *P* < 0.0001; *t*-test).

To investigate the involvement of pSORF3 in the pathogenesis of DPV, we monitored tissue lesions and viral loads in infected animals throughout the experiment. Fourteen-day-old ducklings were inoculated with DPV-ΔSORF3 at a dilution of 10^6^ TCID_50_, while those infected with DPV-CHv50 or DPV-ΔSORF3-Rev served as control groups. The autopsy findings revealed that the experimental animals from all three groups—the parental virus group, rescue virus group, and knockout virus group—exhibited symptoms consistent with systemic sepsis. These included congested and enlarged spleens, hemorrhagic lesions in the proventriculi, congestion and hemorrhage in both the duodena and ceca, and thymic hemorrhage accompanied by atrophy. Notably, during the later stages of viral infection, the thymic size of ducks infected with DPV-ΔSORF3 was restored compared with that of those infected with either DPV-CHv50 or DPV-ΔSORF3-Rev (Fig. 8A and B). We subsequently performed histological examinations on thymus samples. The histology results indicated that thymic cells from the DPV-CHv50 group ducks exhibited severe atrophy, along with increased intercellular space sizes. Conversely, both cellular atrophy and intercellular space formation were significantly reduced in the knockout virus group compared with those in the group infected with the parental strain (Fig. 8C). These findings suggest that the SORF3 protein plays a role in the pathogenesis of DPV; furthermore, the absence of its expression appears to mitigate thymic atrophy induced by this virus. Quantitative assessments of viral copy numbers across various tissues, including the heart, liver, spleen, thymus, duodenum, cecum, proventriculus, and bursa of Fabricius, demonstrated that the deletion of pSORF3 did not significantly impact viral replication in these animal models (Fig. 8D).

### Prediction of pSORF3 function via mass spectrometry

**Fig 9:**
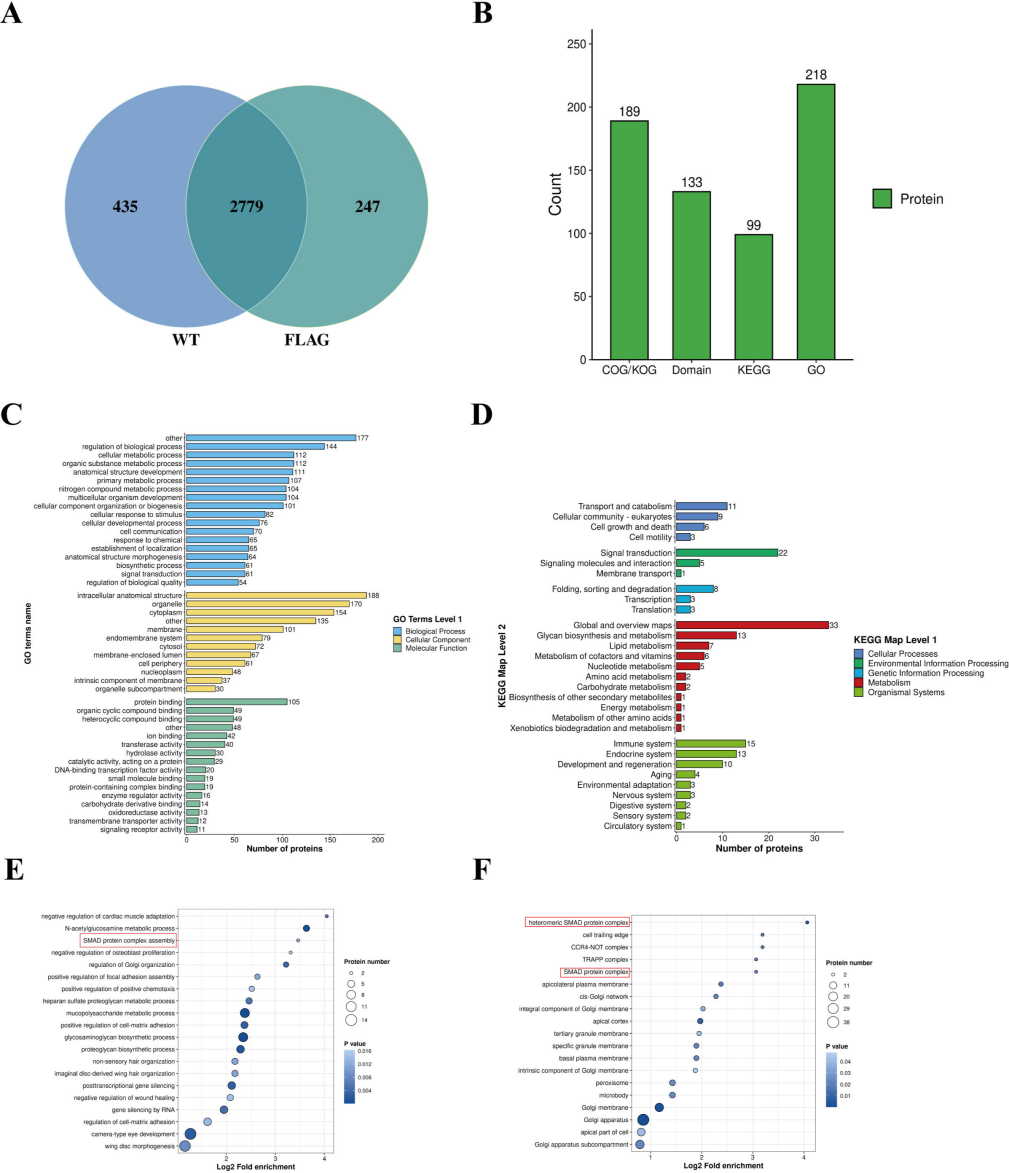
Analysis of the mass spectrometry results. (**A**) A Venn diagram was constructed to illustrate the identified proteins, with the intersection representing the number of proteins common to both sets of MS results. (**B**) The column chart presents the number of proteins that can be annotated by gene ontology (GO), protein domain, KEGG pathway, and COG/KOG functional classifications among the 247 proteins identified through label-free virus mass spectrometry. (**C, D**) The horizontal axis denotes the number of identified proteins within each classification, whereas the vertical axis indicates secondary functional classifications derived from first-level categories. Distinct colors are used in this figure to represent the first-level classifications for GO and KEGG. (**E, F**) The enrichment bubble chart displays the top 20 most significantly enriched functions. In this chart, the vertical axis represents functional descriptions, whereas the horizontal axis illustrates fold enrichment after log_2_ transformation; larger values indicate a higher degree of enrichment. The color of each point corresponds to the *P*-value indicating the significance of enrichment—darker blue shading signifies stronger significance—while the point size reflects the quantity of proteins identified within each GO function; larger points correspond to a greater number of identified proteins.

To investigate the role of the SORF3 protein in the process of DPV infection in DEFs, DEFs were infected with a DPV-BAC-SORF3_C3*Flag_-tagged virus. Proteomic analysis was conducted to identify host proteins that specifically interact with DPV-BAC-SORF3_C3*Flag_ during infection. Similarly, DEFs infected with the wild-type virus served as a control group. The results indicated that infection with both the DPV-BAC-SORF3_C3*Flag_ and WT viruses led to the enrichment of 2,779 proteins, 247 of which were identified as specific to cells infected with DPV-BAC-SORF3_C3*Flag_ (Fig. 9A). To gain deeper insight into the functional characteristics of these different proteins, comprehensive functional annotations were performed on the identified proteins (Fig. 9B and C). Based on the functional enrichment analysis of the specific proteins, SORF3 may be involved in the TGFβ/SMAD signaling pathway (Fig. 9D). However, further research is needed to elucidate precisely how SORF3 functions within this pathway.

## DISCUSSION

The vast DPV genome encodes more than 70 viral proteins, most of which exhibit homology with proteins encoded by other ɑ-herpesviruses. However, research into the impact of these viral proteins on the life cycle and pathogenic mechanisms of DPV remains insufficiently comprehensive. Although some proteins share a high degree of homology, their roles in different pathogens can vary significantly. A notable example is the tegument protein VP16: it is essential for the replication and proliferation of HSV-1, while it is considered non-essential in pseudorabies virus (PRV) and varicella-zoster virus (VZV) (11–15).

In addition, sequencing analyses revealed that certain genes within DPV are unique to avian herpesviruses, including LORF2, LORF3, LORF4, LORF5, and SORF3, which we investigated. As research on the α-herpesvirus genome progresses, the functions of avian herpesvirus-specific genes have become increasingly clear, demonstrating a close connection to viral pathogenicity. For instance, the MDV vLIP gene, an L gene homologous to LORF2, encodes a protein whose lipase activity is abolished by a mutation at position 335. Abrogation of vLIP significantly attenuates both viral replication and pathogenicity (16–18). The DPV LORF3 gene encodes a nuclear late protein that, while dispensable for the essential viral replication cycle, promotes cell-to-cell spread. Its deletion alleviates systemic sepsis and thymic atrophy, highlighting its potential as a vaccine target (9). Although MDV LORF9 is not required for viral replication *in vitro*, it significantly suppresses viral proliferation *in vivo* and reduces pathogenicity and tumorigenesis, identifying it as an important virulence factor (19, 20). The LORF5 gene is essential for MDV replication and associated with virulence, whereas in DPV, it solely facilitates cell-to-cell transmission. This functional divergence is likely attributable to its low sequence conservation and warrants further investigation (21, 22). Collectively, these findings provide a theoretical foundation and novel insights for understanding α-herpesviruses and for future functional studies on SORF3.

Prior to this, research on the SORF3 gene had remained largely unexplored. It remains uncertain whether the specific genes hypothesized are translated into proteins and what their roles are in the viral life cycle. By utilizing the highly virulent strain DPV-CHv50, we demonstrated that the SORF3 protein is produced during the viral infection process, providing the first evidence that the SORF3 gene indeed encodes an independent and novel viral protein. The genes of herpesviruses are expressed in a cascade manner and can be classified into IE genes, E genes, and L genes based on their transcriptional phases (23, 24). Our experimental results indicated that SORF3 belongs to the category of L genes. Notably, when expressed alone, SORF3 is localized entirely within the nucleus; however, its localization shifts to the cytoplasm during viral infection. We speculate that other viral proteins interact with SORF3 during infection, facilitating its translocation from the nucleus to the cytoplasm for functional purposes. This phenomenon is not uncommon among herpesviruses. For example, in HSV-1, UL13 regulates the localization of both UL34 and UL31 by phosphorylating Us3 or through related independent mechanisms (25). Meanwhile, the expression of UL31 itself is both necessary and sufficient for the targeting of the UL34 protein to the nuclear membrane of Hep2 cells (26). Therefore, investigating how SORF3 interacts with other viral proteins, which may lead to its cytoplasmic transfer, has significant implications for understanding its function. To further investigate how SORF3 affects virus replication, we generated a knockout virus that lacks pSORF3 expression. Despite this deletion, our findings indicated that this knockout virus could still infect DEFs and induce a cytopathic effect comparable to that observed with wild-type viruses. The life cycle of herpesviruses encompasses several stages: adsorption, entry, replication of viral nucleic acids, capsid assembly, release of new virions from host cells, and intercellular spread (27). Although the deletion of SORF3 does not affect the adsorption, entry, replication, or release processes of DPV, it significantly weakens the virus’s intercellular transmission capacity and reduces its pathogenicity. This phenotype aligns with the functions of certain conserved genes in α-herpesviruses: in bovine herpesvirus 1, the US4 gene enhances virulence by promoting intercellular spread, and its deletion reduces viral virulence. In infectious laryngotracheitis virus, the deletion of the US4 gene maintains normal replication kinetics but significantly reduces the efficiency of intercellular spread (28, 29). This suggests that SORF3 may enhance DPV pathogenicity by specifically modulating the viral cell-to-cell spread pathway. Undoubtedly, the efficiency of viral transmission plays a critical role in determining virulence (30–33). Finally, to elucidate the biological functions of the SORF3 protein, this study employed high-precision mass spectrometry to identify its interactome systematically. KEGG pathway enrichment analysis revealed significant enrichment of SORF3-interacting proteins in the TGF-β/SMAD signaling pathway. Research has confirmed that the microRNA encoded by the HSV-1 latency-associated transcript inhibits apoptosis in infected cells by modulating TGF-β signaling, thereby promoting latent, persistent infection in sensory neurons (34). Notably, DPV exhibits a highly similar neurotropic latency pattern—specifically colonizing the trigeminal ganglia, maintaining latency through an immunosuppressive microenvironment, and causing characteristic neuropathological damage upon reactivation. Therefore, investigating whether the interaction between the SORF3 protein and the TGF-β/SMAD signaling pathway constitutes a molecular regulatory hub for DPV neural latency could provide insights into the control mechanisms of latent infections across the Herpesviridae family.

In our final exploration, we investigated the impact of this specific novel protein on DPV pathogenesis. The absence of pSORF3 resulted in a reduced mortality rate among the animals. In our study, elimination of the SORF3 protein still led to systemic sepsis and typical “big head disease” symptoms induced by DPV infection. However, notably, pSORF3 appears to mitigate thymic atrophy caused by DPV in ducks. During the development of commercial attenuated vaccines for MDV, researchers have been dedicated to creating an MDV vaccine that does not induce lymphoid organ atrophy in chickens (35). Ensuring that host animal lymphoid organs remain intact may be one of the essential conditions for a safe vaccine. Therefore, whether DPV SORF3 can serve as a target gene for genetically engineered vaccines is a question worthy of further investigation.

## MATERIALS AND METHODS

### Materials

#### Cells and viruses

In this study, all experiments were conducted using DEFs obtained from the farm of Sichuan Agricultural University. DEFs were cultured in DMEM supplemented with 10% newborn bovine serum (NBS; Gibco BRL, Grand Island, NY). Following transfection or infection, the culture medium was replaced with DMEM maintenance medium containing 2% NBS to support continued growth. Wild-type DPV-CHv50 was preserved by the Poultry Disease Prevention and Control Research Center at Sichuan Agricultural University (GenBank accession number JQ647509). The modified viruses DPV-ΔSORF3, DPV-ΔSORF3-Rev, and DPV-BAC-SORF3_C3*Flag_ were constructed using the artificial chromosome rescue platform for recombinant DPV developed in our laboratory.

#### Antibodies

Mouse polyclonal antibody serum against SORF3, rabbit polyclonal antibody serum against UL47, rabbit polyclonal anti-ICP4, and rabbit polyclonal anti-UL29 were all prepared and stored in our laboratory. The following antibodies are commercially available: anti-Flag mouse monoclonal antibody (MBL, Japan) and anti-HA mouse monoclonal antibody (MBL, Japan), both of which were diluted at a ratio of 1:5,000, and horseradish peroxidase (HRP)-labeled affinity-purified goat anti-mouse IgG (ABclonal, China), which was diluted at a ratio of 1:3,000 (fluorescence antibody).

### Method

#### Plasmid construction

The plasmids pET32a(+) and pCAGGS were preserved by the Poultry Disease Prevention and Control Research Center of Sichuan Agricultural University. Using these original plasmids, the SORF3 gene was amplified via PCR and subsequently cloned and inserted into the pET32a and pCAGGS vectors, resulting in the generation of SORF3-His, SORF3-3×Flag, and SORF3-3×HA constructs. The sequences of the primers used are detailed in Table 1.

**TABLE 1:** Primers

Primer	Primer sequence (5′−3′)	Gene
SORF3-Flag-F	gtctcatcattttggcaaagccaccatggctgaaactaategg	pCAGGS-SORF3-3×Flag
SORF3-Flag-R	ccttatcgtcgtcatccttgtaatcaggaactcgtttaacgcgac	pCAGGS-SORF3-3×Flag
SORF3-HA-F	catcattttggcaaagaattcgccaccatggctgaaactaatcggttatt	pCAGGS-SORF3-3×HA
SORF3-HA-R	cacatcataaggataggtaccaggaactcgtttaacgcgac	pCAGGS-SORF3-3×HA
SORF3-His-F	gatatcggatccgaattcatggctgaaactaatcgg	pET32a(+)-SORF3-His
SORF3-His-R	ctcgagtgcggccgcaagcttttaaggaactcgtttaacgcg	pET32a(+)-SORF3-His
ΔSORF3-Kan-F	aagtatttcagagcagtttaggcatacgtttaatatacatagggataacagggtaatcg	SORF3-deleted targeting fragment
ΔSORF3-Kan-R	caagcggtgtcgtgtacatattataaacttttttaatatatgtatattaaacgtatgcctaaactgctctgaaatactttgttacaaccaattaacc	SORF3-deleted targeting fragment
ΔSORF3-F	taatagcgaattggatgacagcaaa	SORF3 deletion identified
ΔSORF3-R	gaccctgaacgcatatatgagaaac	SORF3 deletion identified
SORF3-Rev-F	ttaatcgcaattcttttcaataagtatttcagagcagtttaggcatacgtttaatatacaatggctgaaactaatcggtt	SORF3-returned targeting fragment
SORF3-Rev-R	acatattataaacttttttaatatattaaggaactcgtttaacgc	SORF3-returned targeting fragment
SORF3 Rev-Kan-F	tatattaaaaaagtttataatatgtacacgacaccgcttgtatgtttttatagggataacagggtaatcgat	SORF3-returned targeting fragment
SORF3 Rev-Kan-R	ttgagttttaaattgtttattaaaacattttaaaaacatacaagcggtgtcgtgtacatattataaacttttttaatatagccagtgttacaaccaat	SORF3-returned targeting fragment
ICP4-F	cgttcgctcagctataccct	ICP4
ICP4-R	ggtccgcttatactgagtcca	ICP4
UL29-F	aacctgcgttcgtctccaat	UL29
UL29-R	gtctctctagtcgcatccgc	UL29
UL47-F	aacggagttgcttggagaaca	UL47
UL47-R	tgggcgatgaaacagagtagg	UL47
SORF3-F	ttatttgctgctgcgttta	SORF3
SORF3-R	ccacaggctggttcactt	SORF3
18 s-RNA-F	gtacagtgaaactgcgaatgg	18 sRNA
18 s-RNA-R	cgtcggcatgtattagctcta	18 sRNA
BAC-SORF3-3FLAG-Kan-F	tgtatacgattgggatcaatgtcgcgttaaacgagttcctgattacaaggatgacgacg	SORF3-insert targeting fragment
BAC-SORF3-3FLAG-Kan- R	gcggtgtcgtgtacatattataaacttttttaatatattatttatcatcgtcgtctttatagtcgccagtgttacaaccaatt	SORF3-insert targeting fragment

#### Construction of recombinant viruses

The DPV-ΔSORF3, DPV-ΔSORF3-Rev, and DPV-BAC-SORF3_C3*Flag_ mutant viruses utilized in this study were constructed via a two-step Red recombination system. The construction of DPV-ΔSORF3 and DPV-ΔSORF3-Rev involved the application of the ΔSORF3-Kan-F/R, SORF3-Rev-F/R, and SORF3-Rev-Kan-F/R primers to obtain the ΔSORF3-Kan and SORF3-Rev-Kan fragments through PCR amplification. The virus mutants with ΔMiniF were generated on the pDPV-CHv50 DP recombinant virus artificial chromosome rescue platform. Deletion and complementation of SORF3 were confirmed via PCR amplification using ΔSORF3-F/R primers, RFLP analysis via the restriction endonucleases *EcoR* I and *Kpn* I, and WB. The DPV-BAC-SORF3_C3*Flag_ construct involved the amplification of the SORF3-3×Flag-Kan fragment via PCR using SORF3-3×Flag-F/R and SORF3-3×Flag-Kan-F/R primers. Subsequently, the tagged virus was constructed on the pDPV-BAC-CHv50 DP recombinant virus artificial chromosome rescue platform. The expression of SORF3_C₃*Flag_ was verified through WB.

#### Extraction of RNA and RT-PCR analysis

The quantitative primers ICP4-F/R, UL29-F/R, UL47-F/R, SORF3-F/R, US2-F/R, US10-F/R, and 18 sRNA-F/R were designed and synthesized via Premier 5 (Table 1). Cell samples were collected at various time points (2, 4, 6, 8, 12, 18, 24, 36, 48, and 60 h) following the infection of DEFs with DPV for RNA extraction. Total RNA was isolated via TRIzol reagent according to the following procedure. After the cell suspensions were discarded, 1 mL of ice-cold TRIzol reagent (prechilled at 4°C) was added to each well of a six-well plate, which was subsequently incubated for 5 min on ice. The cells were scraped with sterile cell scrapers, and the lysates were transferred to RNase-free 1.5 mL tubes. After the addition of 200 μL of chloroform, the samples were vortexed vigorously for 15 s and incubated at room temperature (RT) for 5 min. The samples were subsequently centrifuged at 12,000 × *g* for 15 min at 4°C, and the upper aqueous layer was transferred to new tubes. An equal volume of isopropanol was added to precipitate RNA during a 15 min incubation at RT. Following centrifugation at 12,000 × *g* for 10 min at 4°C, the supernatants were discarded, and the pellets were washed twice with 1 mL of 75% ethanol (prepared in DEPC-treated water) via 5 min centrifugation at 7,500 × *g* (4°C). The RNA pellets were air-dried for 15 min at RT, then dissolved in 20 μL–50 μL of DEPC-treated water and quantified immediately via spectrophotometry with A260/A280 purity assessment. Subsequently, reverse transcription was performed to convert the extracted RNA into complementary DNA (cDNA). Real-time fluorescence quantitative PCR was employed to assess the expression levels of each gene. Reactions were performed in 10 μL volumes containing 5 μL of SYBR Green Master Mix (Applied Biosystems), 1 μL of cDNA, 0.3 μL of 10 μM forward primer, 0.3 μL of 10 μM reverse primer (final concentration: 300 nM each), and 3.4 μL of RNase-free water. Each experimental sample was analyzed in triplicate to ensure the reliability of the results; relative transcriptional levels were calculated via the cycle threshold method (2^−△△Ct^).

#### WB

Cell samples were collected at various time points (2, 4, 6, 8, 12, 18, 24, 36, 48, and 60 h) following the infection of DEFs with DPV. The cells were lysed via RIPA buffer, and the resulting lysates were subjected to SDS-PAGE for protein separation. The proteins were subsequently transferred onto PVDF membranes and blocked with a solution of 5% skim milk at RT for 4 h. After the membranes were washed three times with TBS containing Tween-20 (TBST), they were incubated overnight at 4°C with diluted mouse anti-SORF3 polyclonal antibody, rabbit anti-UL47 (dilution: 1:800) polyclonal antibody, rabbit anti-ICP4 (dilution: 1:800) polyclonal antibody, and rabbit anti-UL29 (dilution: 1:800) polyclonal antibody. Following another series of three washes with TBST, the membranes were treated with HRP-conjugated goat anti-rabbit IgG light chain (dilution: 1:3,000) and HRP-conjugated goat anti-mouse IgG secondary antibodies (dilution: 1:3,000) at 4°C for 5 h. After three additional washes with TBST, the protein signals on the PVDF membranes were detected via an enhanced chemiluminescence kit.

#### Drug inhibition test

In this study, CHX and GCV were employed as inhibitors of protein synthesis and nucleic acid synthesis, respectively, to ascertain the gene type of SORF3. DEFs cultured in 12-well plates were treated with 250 μg/mL CHX or GCV prior to infection with DPV at an MOI of 0.01. Following incubation at 37°C for 18 h, total RNA was extracted from the cells and subsequently reverse-transcribed into cDNA. PCR was conducted using cDNA as the template, and the resulting PCR products were analyzed via electrophoresis on a 1% agarose gel.

#### Indirect immunofluorescence

Fibroblasts from duck embryos were prepared on coverslips and inoculated with the virus. The cell coverslips were harvested at 2, 4, 6, and 8 h postviral inoculation. The cells were fixed with 4% paraformaldehyde for 15 min at RT, permeabilized with 0.2% Triton X-100 for 10 min at RT, and blocked with 5% BSA for 30 min at 37°C. The samples were then incubated with an anti-FLAG mouse monoclonal primary antibody (1:200 dilution) overnight at 4°C, washed three times with PBS, and stained with an Alexa Fluor 488-conjugated goat anti-mouse IgG secondary antibody (1:500 dilution) for 1 h in the dark. After nuclear counterstaining with DAPI, the coverslips were mounted with antifade reagent and imaged via a confocal laser scanning microscope.

#### *In vitro* growth kinetics

DEFs were cultured in 24-well plates and subsequently infected with DPV-CHv50, DPV-ΔSORF3, or DPV-ΔSORF3-Rev at an MOI of 0.01. Following a 2 h incubation at 37°C, the medium was replaced with maintenance medium supplemented with 2% NBS. Samples of infected cells, supernatants, and total solutions were collected at intervals of 12, 24, and 48 h, as well as at final time points of 72 h and 96 h; the total volume of each sample was adjusted to a final volume of 500 μL. The viral titers were assessed by measuring the TCID_50_ of the virus in the cells, supernatants, and total solution.

#### Real-time fluorescence quantitative PCR

Viral DNA was extracted from the samples via a Taigen Blood/Cell/Tissue Genomic DNA Extraction Kit (DP304; Taigen; Beijing, China) and subsequently analyzed via quantitative PCR (qPCR). The DPV UL30-specific primers and probes, previously designed in our laboratory, are detailed in Table 1. The copy number of viral DNA was quantified via Ex Taq premix (probe qPCR) (TaKaRa, Dalian, China). The qPCR amplification protocol consisted of initial denaturation at 95°C for 30 s, followed by 40 cycles of denaturation at 95°C for 5 s and annealing/extension at 60°C for 30 s. The qPCR results were quantified by comparing them with a standard curve established in our laboratory. All reactions were performed in triplicate, and a minimum of three independent experiments were conducted to ensure reliability.

#### Adsorption, entry, replication, and release of the virus

Adsorption: DEFs were precooled at 4°C for 1 h, infected with DPV-CHv50, DPV-ΔSORF3, or DPV-ΔSORF3-Rev, and incubated at 4°C for an additional 2 h to facilitate binding while preventing internalization of the virus. The cell samples were subsequently washed five times with precooled PBS, and DMEM was added. After performing freeze-thaw cycles, the viral genome was extracted from the samples to determine the virus copy number. Entry: Following inoculation with the three viruses, the DEFs were precooled at 4°C for 1 h and then incubated at this temperature for an additional 2 h. The culture medium was then replaced, and the temperature was increased to 37°C to allow viral entry. Three hours after temperature adjustment, the cell samples were collected, and viral DNA was extracted for quantification. Replication: DEFs were incubated with a recombinant virus at an MOI of 0.05 at 37°C for 6 h; thereafter, the culture medium was changed to 2% NBS-DMEM. The cell samples were collected at intervals of 6 h to 10 h after the culture medium was changed for fluorescence quantitative analysis. Release: DEFs were incubated with a recombinant virus at an MOI of 0.05 under similar conditions as above (37°C for 6 h), followed by replacement of the culture medium with 2% NBS-DMEM. After an incubation period of 18 h following medium change, cell supernatants were collected every 60 min up to 400 min to assess TCID_50_ levels.

#### Virus plaque assay

Cellular plaques were visualized via crystal violet staining. The intercellular spread of each mutant virus was assessed by measuring the resulting plaque size. DEF cells in six-well plates were infected with each mutant virus at an MOI of 0.001. Following incubation at 37°C for 2 h, a 1.5% methylcellulose solution (Solarbio, Beijing, China) was used to overlay the cells. Five days post-infection, crystal violet staining was used to visualize the cellular plaques. The cells were fixed with 500 μL of precooled 4% paraformaldehyde for 20 min, washed twice with sterile PBS, and then stained with 500 μL of 0.5% crystal violet for an additional 30 min. The cells were subsequently rinsed with tap water to remove excess stain, after which the plaques were observed and counted. The average plaque size was quantified via ImageJ software (NIH, Bethesda, MD, USA). The plaque sizes of both the mutant virus and restored virus were calculated and compared with that of the parental virus, which was set as a reference value at 100%. A *P*-value threshold of <0.05 indicated statistical significance (***) when *P* < 0.001.

#### Animal experiments

To investigate the role of SORF3 in the replication and pathogenesis of DPV in infected ducks, 10^6^ TCID_50_ of the parental wild-type DPV-CHv50 was intramuscularly administered to 14-day-old ducklings (*n* = 10). Additionally, groups were established for DPV-ΔSORF3-Rev (*n* = 10) and DPV-ΔSORF3 (*n* = 10) infection. The clinical symptoms exhibited by the ducklings, including rectal temperature, body weight, mortality rate, ataxia, paralysis, torticollis, lethargy, and other relevant signs, were monitored daily. Throughout and following the experiment, histopathological examinations were performed on those ducklings that died from disease or were humanely euthanized.

#### Determination of viral loads in organs and tissues

On the 2nd, 5th, and 10th days post-infection, hearts, livers, spleens, proventriculi, duodena, ceca, bursae of Fabricius, and thymuses from infected animals in each group were collected. DNA was extracted from each organ and tissue using a blood, cell, and tissue genomic DNA extraction kit (Qiagen; DP304) according to the manufacturer’s instructions. As previously mentioned, the DPV genome copy number was quantified via qPCR.

#### LC-MS/MS analysis

DEF cells were infected separately with CHv50 (WT) and DPV-BAC-SORF3_C3*Flag_ (Flag) at an MOI of 1. At 36 hpi, cell lysates were harvested and incubated with Anti-Flag affinity beads. The bead-bound complexes were subsequently subjected to mass spectrometry identification. The mass spectrometry identification and proteomics analysis services were provided by PTM Biolabs, Inc. During result analysis, proteins detected only in the DPV-BAC-SORF3_C3*Flag_ group, but not in the CHv50 (WT) group were identified as specific binding partners of the SORF3 protein; proteins detected in both experimental groups were considered non-specific binding proteins.

#### Statistical analysis

Statistical analysis was conducted via GraphPad Prism version 8 (San Diego, CA, USA). A *P*-value of less than 0.05 was considered indicative of a statistically significant difference in the data. The growth kinetics and plaque size data were analyzed via one-way analysis of variance.

## Data Availability

The data supporting the findings of this study are available in the Figshare repository (https://info.figshare.com/, accession number: https://doi.org/10.6084/m9.figshare.30730319) and are also available from the corresponding author upon reasonable request. The GenBank accession number for the SORF3 gene of DPV used in this study is AFC61890.1.
